# Supplementary material for: A Naked Lyophilized mRNA Vaccine Against Seasonal Influenza, Administered by Jet Injection, Provides a Robust Response in Immunized Mice
Source: Vaccines (Basel). 2026 Jan 2;14(1):56. doi: 10.3390/vaccines14010056 (PMC12846339; doi:10.3390/vaccines14010056)
Supplement: Supplementary file 1 [file vaccines-14-00056-s001.zip › vaccines-4016513-supplementary.pdf]

Supplemental Figures

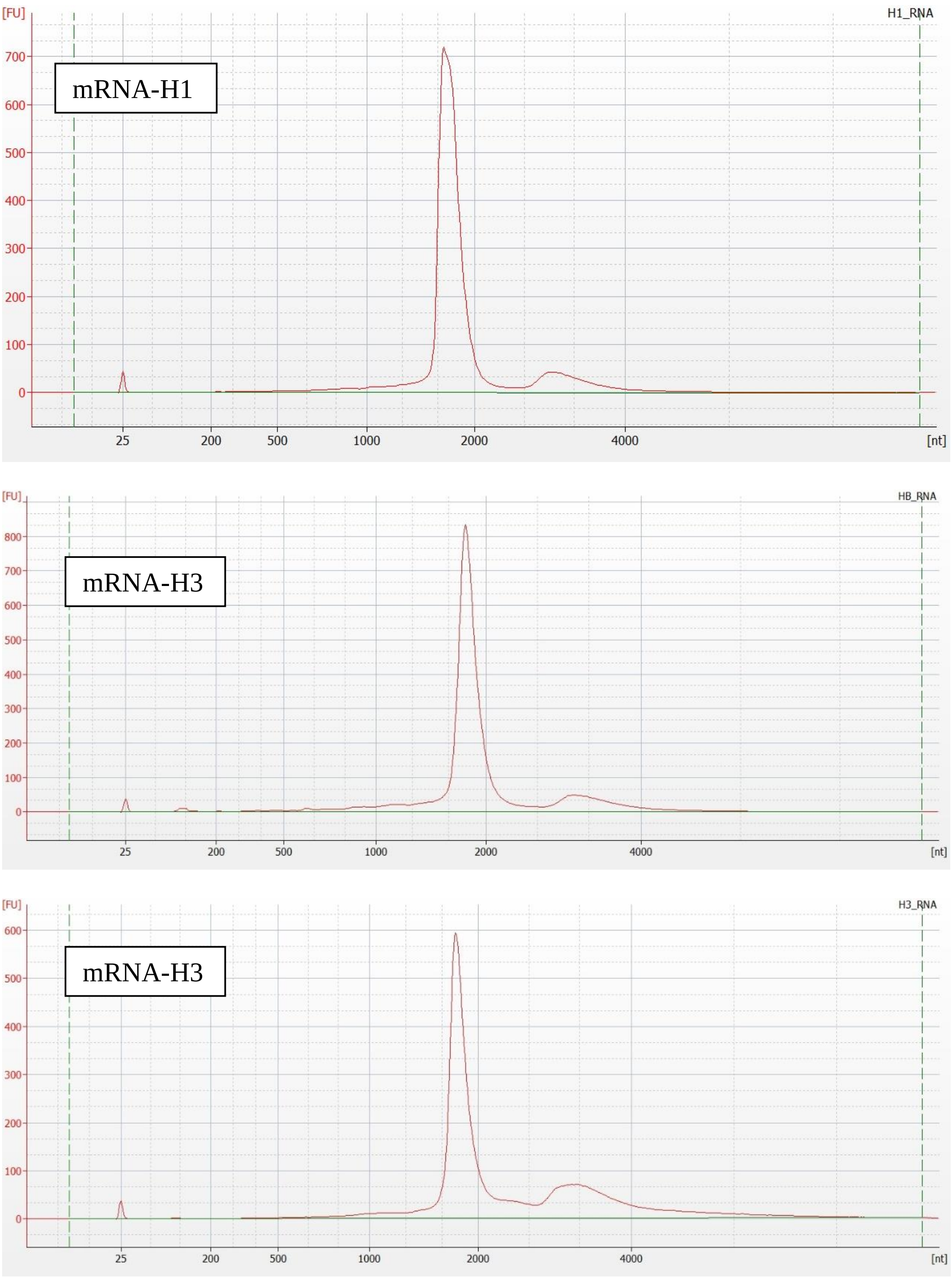

Figure S1. Electropherogram of synthesized mRNA-H1, mRNA-H3 and mRNA-HB obtained by capillary electrophoresis

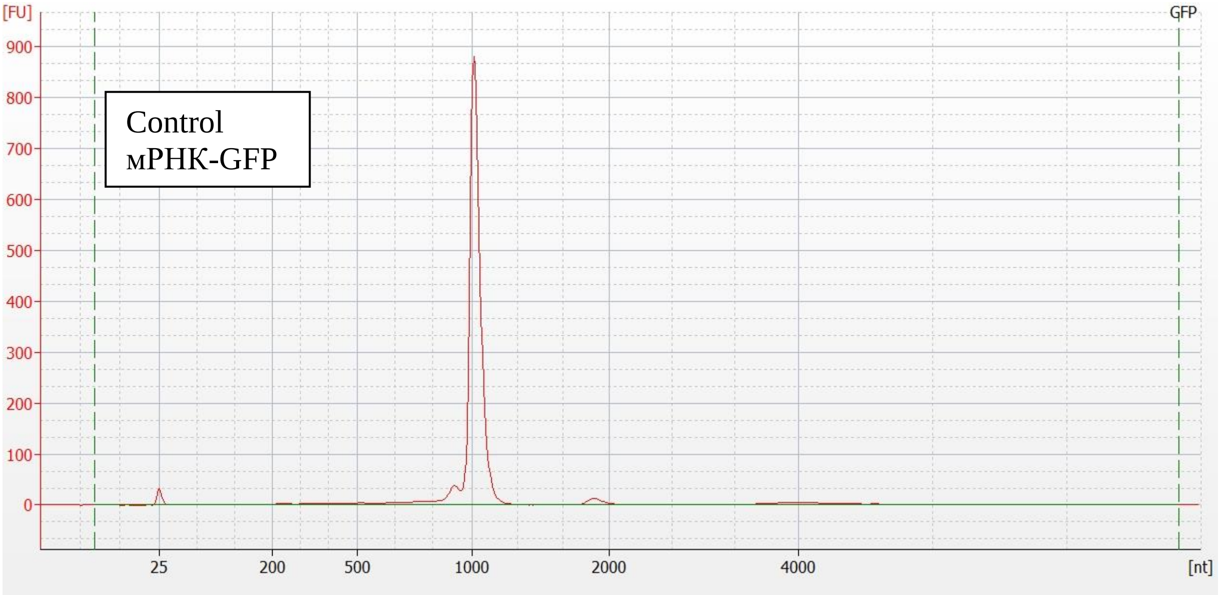

Control
мРНК-GFP
[FU]
900
800
700
600
500
400
300
200
100
0
25
200
500
1000
2000
4000
[nt]
GFP

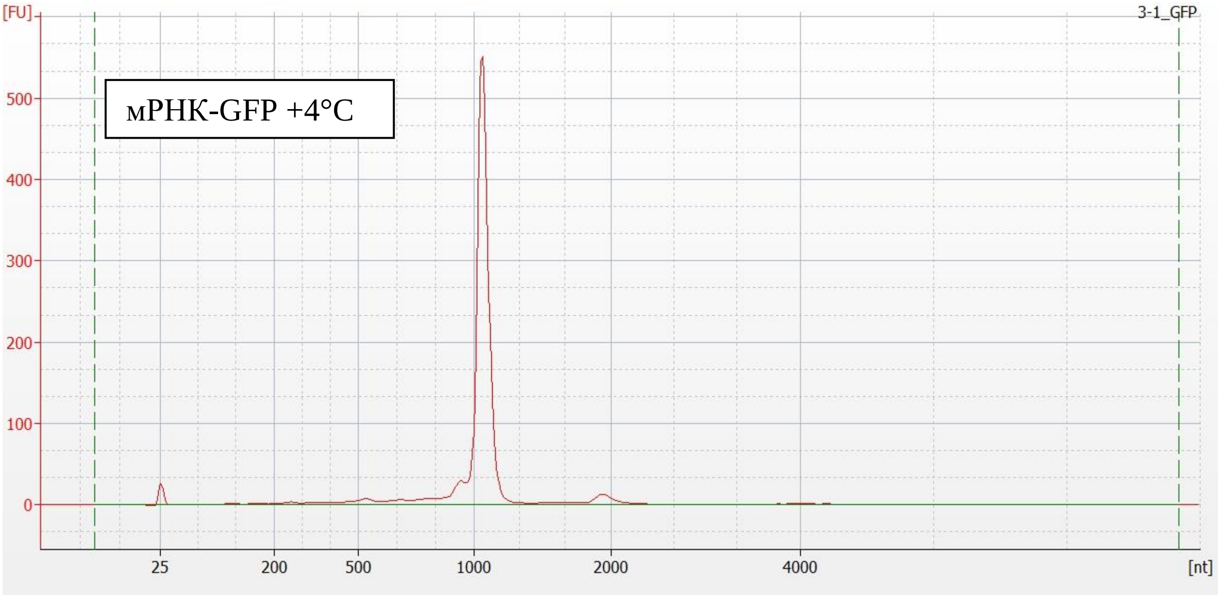

мРНК-GFP +4°C
[FU]
500
400
300
200
100
0
25
200
500
1000
2000
4000
[nt]
3-1_GFP

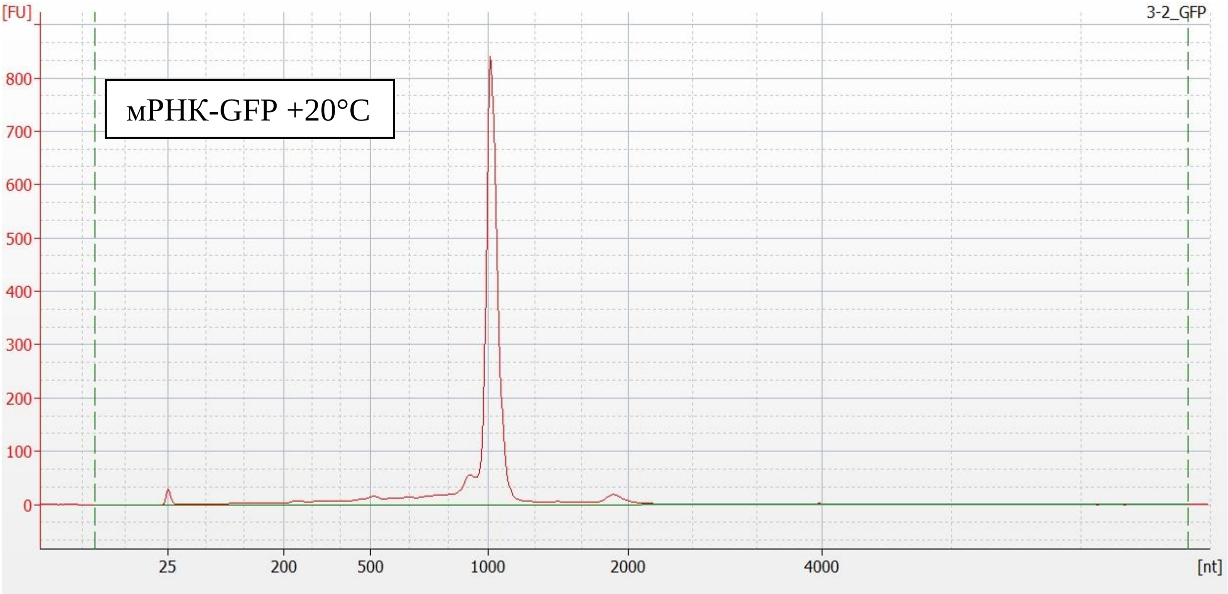

мРНК-GFP +20°C
[FU]
800
700
600
500
400
300
200
100
0
25
200
500
1000
2000
4000
[nt]
3-2_GFP

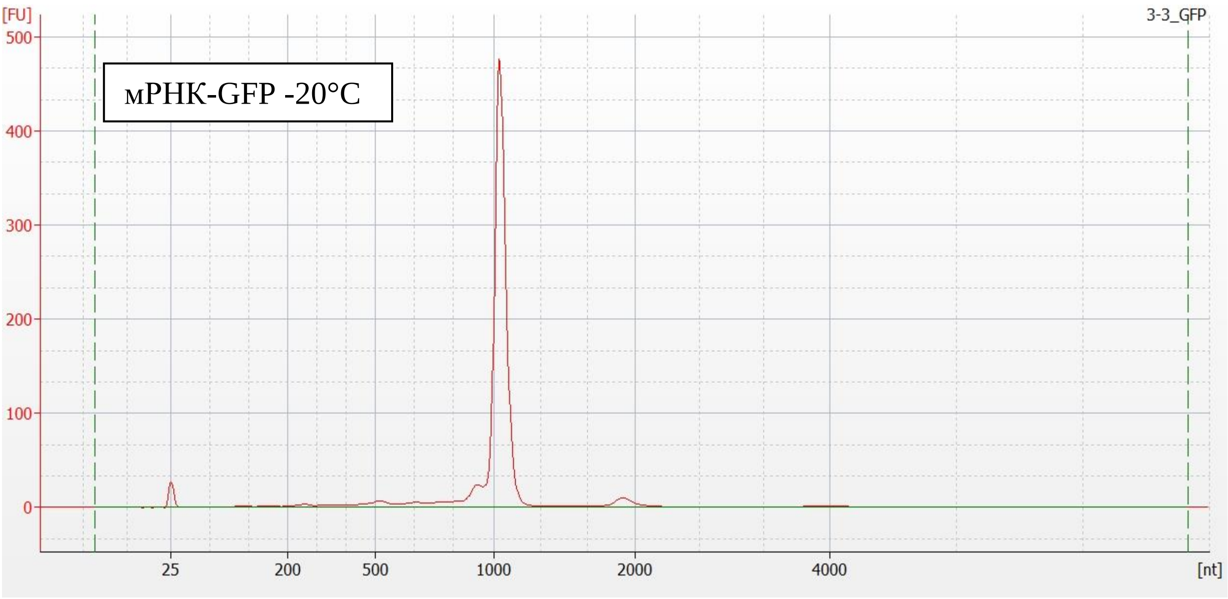

Figure S2. Electropherogram of lyophilized mRNA-GFP stored for 3 months, obtained by capillary electrophoresis. Control mRNA-GFP - synthesized immediately before use mRNA-GFP; mRNA-GFP +4°C - lyophilized mRNA-GFP stored for 3 months at +4°C; mRNA-GFP +20°C - lyophilized mRNA-GFP stored for 3 months at +20°C; mRNA-GFP -20°C - lyophilized mRNA-GFP stored for 3 months at -20°C;

<table>
<tr><td></td><td>Control, synthesized immediately before use mRNA-GFP</td><td>mRNA-GFP stored at +4°C</td><td>mRNA-GFP stored at +20°C</td><td>mRNA-GFP stored at -20°C</td></tr>
<tr><td>1 month storage</td><td>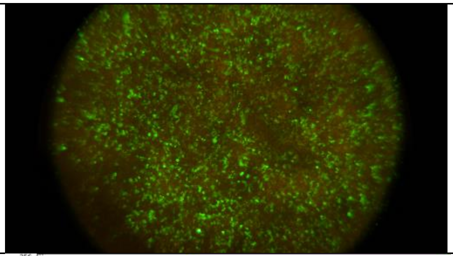</td><td>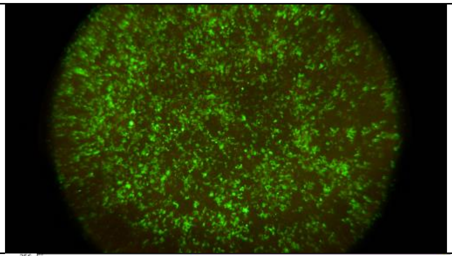</td><td>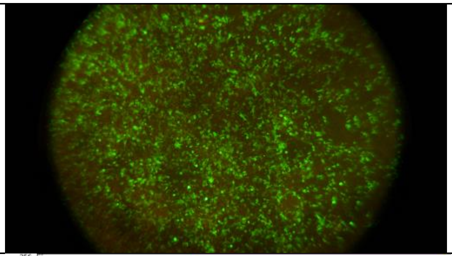</td><td>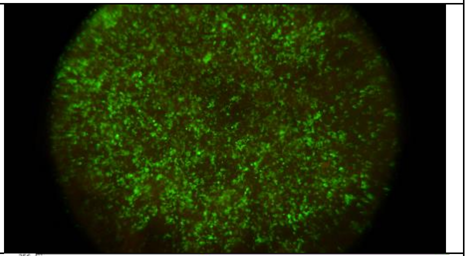</td></tr>
<tr><td></td><td>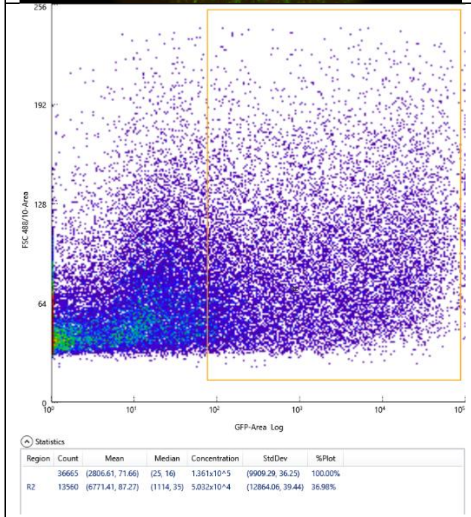

36,9%</td><td>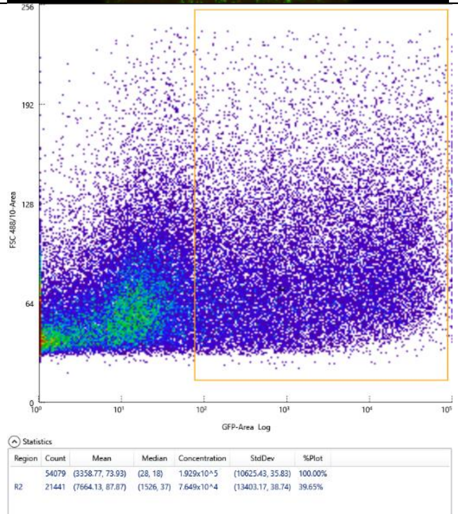

39,65%</td><td>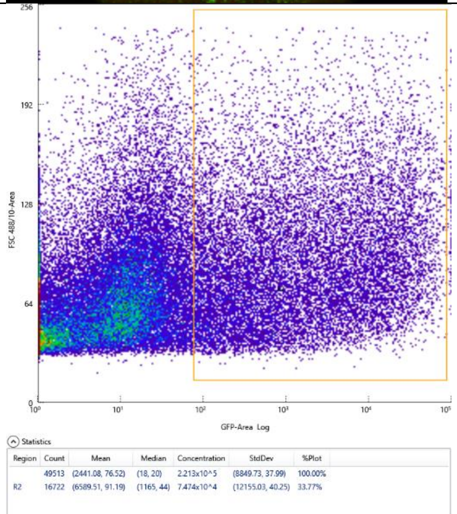

33,77%</td><td>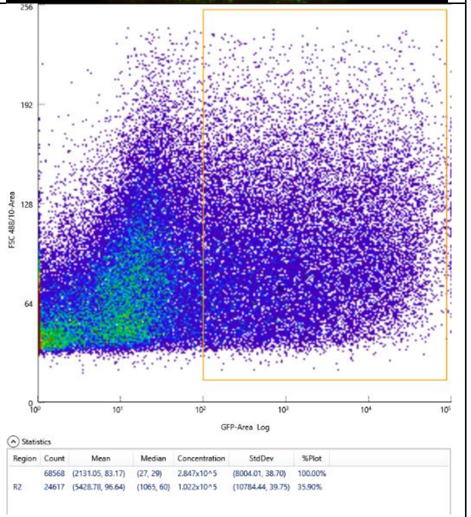

35,9%</td></tr>
<tr><td>3 month storage</td><td>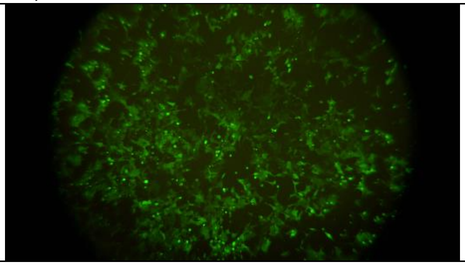</td><td>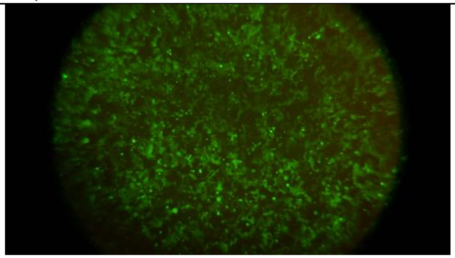</td><td>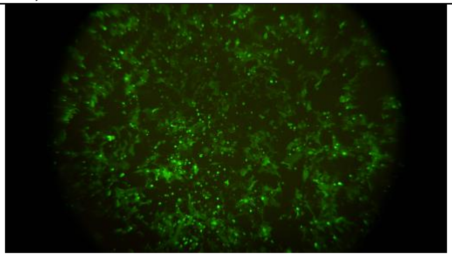</td><td>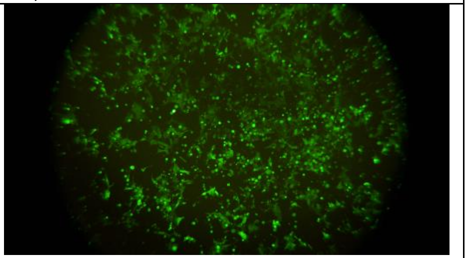</td></tr>
</table>

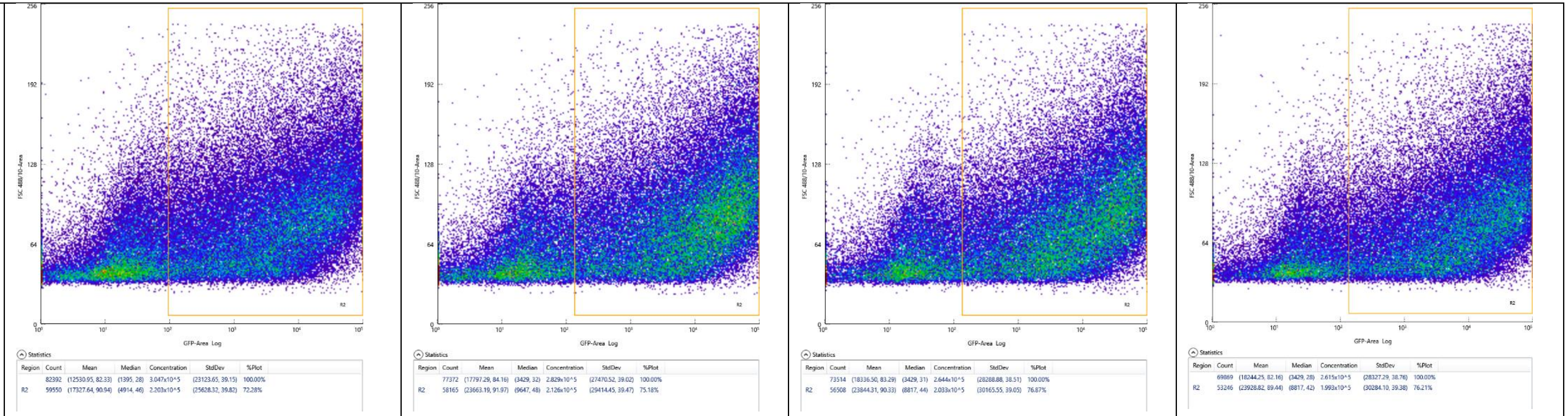

72,28% | 75,18% | 76,8% | 76,21%

Figure S3. Evaluation of the efficiency of GFP protein synthesis in HEK293 cells transfected with lyophilized GFP mRNA. Micrograph of HEK293 cells transfected with different GFP mRNA preparations and flow cytometry data.

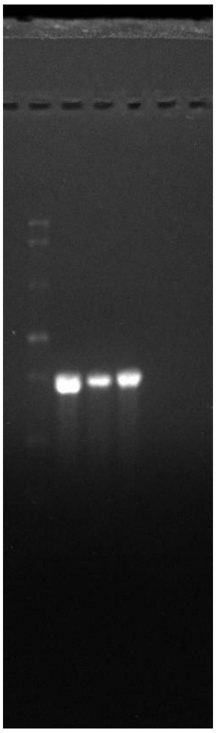

Figure S4. Image of the original electrophoresis 1b.

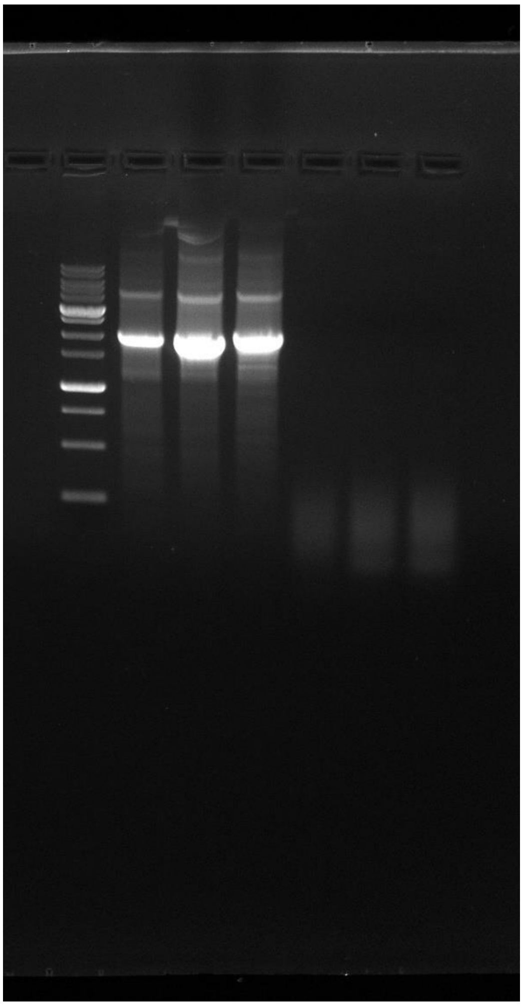

Figure S5. Image of the original electrophoresis 1c.
